# Epidemiology and clinical characteristics of hospitalized patients with pandemic influenza A (H1N1) 2009 infections: the effects of bacterial coinfection

**DOI:** 10.1186/1743-422X-8-501

**Published:** 2011-11-03

**Authors:** Amreeta Dhanoa, Ngim C Fang, Sharifah S Hassan, Priyatharisni Kaniappan, Ganeswrie Rajasekaram

**Affiliations:** 1 Jeffrey Cheah School of Medicine and Health Sciences, Monash University, Malaysia; 2 Johor Bahru Clinical School, Monash University, Malaysia; 3 Department of Pathology, Johor Bahru General Hospital, Malaysia

**Keywords:** Bacterial coinfection, H1N1, hospitalized, neutrophilia, complications, age

## Abstract

**Background:**

Numerous reports have described the epidemiological and clinical characteristics of influenza A (H1N1) 2009 infected patients. However, data on the effects of bacterial coinfection on these patients are very scarce. Therefore, this study explores the impact of bacterial coinfection on the clinical and laboratory parameters amongst H1N1 hospitalized patients.

**Findings:**

This retrospective study involved hospitalized patients with laboratory-confirmed H1N1 infections (September 2009 to May 2010). Relevant clinical data and the detection of bacterial coinfection from respiratory or sterile site samples were obtained. Multiplex PCR was used to determine the co-existence of other respiratory viruses. Comparison was made between patients with and without bacterial coinfection. The occurrence of coinfection was 34%; 14 (28%) bacterial and only 3 (6%) viral. *Mycoplasma pneumoniae *(n = 5) was the commonest bacteria followed by *Staphylococcus aureus *(n = 3). In univariate analysis, clinical factors associated with bacterial coinfection were age > 50 years (p = 0.02), presence of comorbidity (p = 0.04), liver impairment (p = 0.02), development of complications (p = 0.004) and supplemental oxygen requirement (p = 0.02). Leukocytosis (p = 0.02) and neutrophilia (p = 0.004) were higher in bacterial coinfected patients. Multivariate logistic regression analysis revealed that age > 50 years and combined complications were predictive of bacterial coinfection.

**Conclusions:**

Bacterial coinfection is not uncommon in H1N1 infected patients and is more frequently noted in the older aged patients and is associated with higher rates of complications. Also, as adjunct to clinical findings, clinicians need to have a higher index of suspicion if neutrophilia was identified at admission as it may denote bacterial coinfection.

## Background

In April 2009, a novel influenza A (H1N1) virus emerged in Mexico and spread rapidly worldwide [1]. By June 11, 2009 nearly 30, 000 cases had been confirmed across 74 countries including Malaysia, prompting World Health Organization to raise its pandemic alert to phase 6 [2]. After the first reported H1N1 case in Malaysia in May 15, 2009, the numbers increased exponentially and as of May 31, 2010 they totaled 14, 821 with 87 deaths [3]. Thereafter, there have been numerous reports describing the epidemiological and clinical characteristics of H1N1 infections. However, studies focusing on the effects of respiratory pathogen coinfection on clinical and laboratory parameters in the H1N1 infected patients are scarce. Clinicians may assume that a single virus type is involved, as laboratory detection involves PCR specifically targeting H1N1. However, bacterial coinfection had been shown to contribute to morbidity and mortality in previous influenza pandemics [4]. Therefore, this study aims to explore the clinical and laboratory characteristics amongst patients hospitalized with laboratory-confirmed pandemic influenza A (H1N1) infection and the effects of bacterial coinfection on these parameters.

## Findings

### Methodology

This retrospective study was conducted from September 2009 to May 2010 at Hospital Sultanah Aminah Johor Bahru (HSAJB). HSAJB is a 989-bedded tertiary referral centre and the government designated hospital for H1N1 testing in Johor State, Malaysia. As the main General Hospital of Johor, its' patient population is reflective of the larger community in Malaysia. During our study period, which coincided with the peak of H1N1 pandemic activity, all patients regardless of whether they were hospitalized or not, who presented with an influenza-like illness (ILI) were tested for H1N1.

Consecutive hospitalized patients with laboratory-confirmed H1N1 infections were identified from microbiology laboratory records. Laboratory diagnosis of H1N1 was made using the Centers for Disease Control and Prevention (CDC) real-time reverse transcriptase polymerase chain reaction (RT-PCR) protocol [5]. Relevant clinical data was retrieved from patients' medical records. The presence of bacterial coinfection from respiratory specimens (sputum, tracheal/nasopharyngeal aspirate, bronchoalveolar lavage) or sterile site samples (blood or pleural fluid) taken within 48 hours of admission was recorded. *Mycoplasma pneumoniae *infection was diagnosed by serology using particle agglutination test (Serodia- Myco II, Fujirebio Inc., Japan). A single titer of ≥ 160 was considered as diagnostic cut-off titer, based on population background study conducted in Malaysia [6,7]. All samples confirmed H1N1 positive were stored at -80°C for further analysis using multiplex PCR (Seeplex RV Detection, USA) which detects adenovirus, influenza virus A and B, respiratory syncytial virus, parainfluenza types 1, 2 and 3 and human metapneumovirus. Hematological, liver and renal function parameters on admission were recorded.

Data was analyzed using SPSS version 17.0.1; comparing patients with and without bacterial coinfection with a *P*-value < 0.05 (two-tailed) taken as the level of significance. Variables associated with bacterial coinfection in the univariate analysis were then entered into multivariate logistic regression analysis.

## Results

After excluding 7 patients (5 incomplete data and 2 for presence of nosocomial pneumonia), data of 50 patients was available for analysis (Table 1). The patients age ranged from 7 months to 82 years (median 20.3 years), with 90% patients (45/50) < 50 years. Excluding 6 pregnancies, 24 patients (48%) had at least one preexisting comorbidity; lung disease being the commonest. The mean duration of symptoms before hospitalization was 4.4 ± 3.08 days (range 1-14 days). Cough (100%) and fever (98%) were the most common symptoms on admission. Twelve patients (24%) had oxygen saturation < 95% at presentation. Pneumonia was diagnosed in 25 patients (50%) based on clinical and radiological findings.

**Table 1:** Clinical and laboratory characteristics of hospitalized patients with pandemic influenza A (H1N1) 2009 infections

Characteristics	No of patients (N = 50)	%
Male sex	25	50
Age < 50	45	90
Paediatric (≤ 15 years)	23	46
Cough	50	100
Fever	49	98
Dyspnoea	24	48
Rhinorrhoea	24	48
Sore throat*	16	36.4
Vomiting	12	24
Myalgia*	7	15.9
Headache*	7	15.9
Hypoxaemia	12	24
Tachypnoea	16	32
Pneumonia	25	50
Comorbidity**	24	48
Lung disease^¶^	11	22
Hypertension	5	10
Diabetes mellitus	5	10
Malignancy ^Ψ^	3	6
Autoimmune^¥ ^	2	4
Others ^£^	6	12
Complications^® ^**	13	26
Pregnant	6	12
Coinfection **^Ω ^**	17	34
Leukopenia^©^	4	8
Lymphopaenia^©^	31	62
Leukocytosis^©^	12	24
Neutrophilia ^©^	13	26
ALF ^§ ^	12	29.3
ARF ^§^	4	9.5
ICU	9	18
Supplemental oxygen	22	44
Mechanical ventilation	6	12
Died	2	4

* Not assessed in children < 3 years (n = 44)

** A patient may have more than one comorbidity or complications

¶ Includes asthma (n- = 9), Chronic obstructive airway disease (n = 1) and bronchiectasis (n = 1)

Ψ Includes chronic myeloid leukemia (n = 1), acute myeloid leukemia (n = 1), meningioma of brain (n = 1)

¥ Includes autoimmune haemolytic anaemia (n = 1), idiopathic thrombocytopenia purpura (n = 1)

£Includes cardiovascular disease (n = 2), immunosuppressives (n = 2), hypothyroidism (n = 1), stroke (n = 1)

**^® ^**Includes liver impairment (n = 12), renal impairment (n = 4) septic shock (n = 2) and ARDS (n = 2).

Ω Includes bacterial (n = 14), viral (n = 3). The sites for isolation of 9 non-*Mycoplasma *bacteria: (blood = 2, sputum = 3, nasopharyngeal aspirate = 3, bronchoalveolar lavage = 2)

^© ^Established values in our laboratory, Adults: leukocytes 4-11 × 10^9^/L; neutrophils 2-7.5 × 10^9^/L; lymphocyte 1.5-4 × 10^9^/L; Paediatrics: age-dependent

ALF: abnormal liver function (n = 41) (raised alanine aminotransferase/aspartate aminotransferase or both)

ARF: abnormal renal function (n = 42) (raised creatinine)

All patients received oseltamivir after admission. Twenty-two patients (44%) required oxygen supplementation. Nine cases (18%) were treated at the intensive care unit (ICU); 6 requiring mechanical ventilation. Thirteen patients (26%) developed complications (single or combination); liver impairment (n = 12), renal impairment (n = 4) septic shock (n = 2) and acute respiratory distress syndrome (ARDS) (n = 2). Two (4%) patients died, resulting from septicaemic shock and severe pneumonia respectively.

### Impact of Bacterial Coinfection

Forty-five patients (90%) had lower respiratory tract specimens sent for bacterial cultures. The 5 patients without these specimens were children who had difficulty in producing respiratory secretions, however, they appeared generally well with no evidence of pneumonia. Blood cultures were performed in 23 patients (46%) and *Mycoplasma pneumoniae *serology in 27 patients (54%). Of the 50 H1N1 patients, 17 (34%) were coinfected with a second respiratory pathogen; 14 (28%) bacterial and only 3 (6%) viral. *Mycoplasma pneumoniae *(n = 5) was the commonest bacterial coinfection followed by *Staphylococcus aureus *(n = 3), *Klebsiella pneumoniae *(n = 2), *Streptococcus pneumoniae *(n = 2), *Moraxella catarrhalis *(n = 1)*, Pseudomonas aeruginosa *(n = 1), *Streptococcus pyogenes *(n = 1) and *Streptococcus agalactiae *(n = 1). Two patients had dual infection; *M.pneumoniae*/*S.agalactiae *and *S.pneumoniae*/M.catarrhalis respectively. The sites for isolation of 9 non-*Mycoplasma *bacteria were blood (2), sputum (3), nasopharyngeal aspirate (3) and bronchoalveolar lavage (2). The 3 virus detected were parainfluenza; these 3 patients presented with influenza-like illness with no deterioration of clinical findings.

A comparison between H1N1 patients with and without bacterial coinfection is shown in Table 2. Although 90% of patients were < 50 years old, bacterial coinfection was more frequent in patients > 50 years (p = 0.02). The presence of underlying comorbidity provided a suitable niche for bacterial coinfection (p = 0.04). Although ICU admissions, mechanical ventilation, renal impairment, mortality and pneumonia were notably higher in patients with bacterial coinfection, they were not statistically significant. Other factors associated with bacterial coinfection in the univariate analysis were development of complications (p = 0.004), liver impairment (p = 0.02) and supplemental oxygen requirement (p = 0.02). Out of the 50 patients, 12 (24%) had leukocytosis and 13 (26%) neutrophilia. Bacterial coinfected patients demonstrated higher rates of leukocytosis (p = 0.02) and neutrophilia (p = 0.004). On the other hand, lymphopenia (n = 31) was notably higher in single viral H1N1 infection. Multivariate analysis revealed that age > 50 (OR 12.577; 95% CI 1-165.24; p = 0.05)) and development of complications (OR 9.01; 95% CI 1.70-47.67; p = 0.01) were predictive of bacterial coinfection.

**Table 2:** Comparison between hospitalized pandemic influenza A (H1N1) patients with or without bacterial coinfection

Variable	All patients n(%)/median(± IQR) N = 50	Bacterial coinfection n(%)/median(± IQR)		P	OR	95% C1
		Yes (n = 14)	No (n = 36)			
Male ^b^	25(50)	7(50.0)	18(50.0)	1.00	1.00	0.29-3.44
Age > 50 ^a ^	5(10)	4(28.6)	1(2.8)	0.02	14.00	1.40-139.81
Paediatric ^b^	23(46)	7(50.0)	16(44.4)	0.72	1.25	0.36-4.31
Dyspnoea ^b^	24(48)	7(50.0)	17(47.2)	0.86	1.12	0.33-3.84
Hypoxaemia ^a^	12(24)	5(35.7)	7(19.4)	0.28	2.30	0.59-9.06
Comorbidity ^b^	24(48)	10(71.4)	14(38.9)	0.04	3.93	1.03-15.00
Diabetes mellitus ^a^	5(10)	3(21.4)	2(5.6)	0.13	4.64	0.68-31.44
Hypertension ^a^	5(10)	3(21.4)	2(5.6)	0.13	4.64	0.68-31.44
Lung disease ^a^	11(22)	4(28.6)	7(19.4)	0.48	1.66	0.40-6.88
Pregnancy^a^	6(12)	1(7.1)	5(13.9)	0.66	0.48	0.05-4.50
Leukopenia ^a^	4(8)	1(7.1)	3(8.3)	1	0.85	0.08-8.89
Lymphopaenia ^b^	31(62)	6(42.9)	25(69.4)	0.08	0.33	0.09-1.18
Leukocytosis ^a^	12(24)	7(50.0)	5(13.9)	0.02	6.20	1.51-25.41
Neutrophilia ^a^	13(26)	8(57.1)	5(13.9)	0.004	8.27	2.00-34.16
Liver impairment ^a^	12(29.3)	7(53.8)	5(17.9)	0.02	6.72	1.50-30.07
Renal impairment ^a^	4(9.5)	3(25.0)	1(3.3)	0.06	9.67	O.89-104.82
Supplemental oxygen ^b^	22(44)	10(71.4)	12(33.3)	0.02	5.00	1.30-19.30
Mechanical ventilation ^a^	6(12)	3(21.4)	3(8.3)	0.33	3.00	0.53-17.09
ICU stay ^a^	9(18)	4(28.6)	5(13.9)	0.25	2.48	0.56-11.07
Pneumonia ^b^	25(50)	9(64.3)	16(44.4)	0.21	0.44	0.12-1.60
Complications ^a^	13(26)	8(57.1)	5(13.9)	0.004	8.27	2.00-34.16
Died ^a^	2(4)	2(14.3)	0(0)	0.07	0.25	0.15-0.41
Antibiotics on admission ^a ^	41(82)	14(100)	27(75.0)	0.05	1.52	1.22-1.89
Antibiotics pre-admission ^a^	8(16)	3(21.4)	5(13.9)	0.67	1.70	0.35-8.28
Duration of hospitalization^c^	5(3)	5(3.5)	5(3.25)	0.25	Non applicable	

^a ^Fisher's exact test; ^b^Chi-squared test; ^c^Non-parametric Mann-Whitney test.

^a ^and ^b ^expressed as number (%); ^c ^expressed as median (± IQR)

N = 50 for all patients except liver impairment n = 41 and renal impairment n = 42

Forty-one patients (82%) received antibiotics, either as empiric or definitive therapy upon admission and 16% prior to admission All patients with bacterial coinfection were treated with antibiotics; significantly higher rates compared to patients without bacterial coinfection (p = 0.05).

## Discussion

The bacterial coinfection rate of 28% amongst our H1N1 hospitalized patients was higher compared to other studies [8,9]. A large laboratory-based study in the United States demonstrated comparable bacterial coinfection rates to our study with similarly very low frequency of viral copathogen detection [10]. Whilst our finding concurred with several studies [1,8,9,11,12] that showed H1N1 infections having a predilection for younger patients, patients > 50 years had higher risk of bacterial coinfection in our study.

Although concurrent bacterial infection was shown to have a major influence on mortality in previous influenza pandemics [4], its' role in the current H1N1 pandemic is still evolving. Recent postmortem studies amongst fatal H1N1 cases established a link between bacterial lung infections and increased deaths [13]. Whilst an earlier study showed bacterial coinfection not to be a major contributor to severe disease [12], a more recent study demonstrated otherwise [8]. In our study, patients with bacterial coinfection were found to have higher risk of developing complications. The presence of underlying comorbidity, liver impairment and supplemental oxygen requirement were significantly higher in bacterial coinfected patients in univariate analysis, although these factors were not predictive in multivariate analysis.

Unlike S.pneumoniae, S.aureus and *S.pyogenes *which are repeatedly reported as coinfecting agents [4,8,10,13], the high rates of *M.pneumoniae *coinfection was unique to our study. Although hematological parameters have been mentioned in few other studies [8,9,12], to our best knowledge this is the first study that specifically explored the impact of bacterial coinfection on these parameters. CDC recognizes the importance of early empirical antibiotics in H1N1 infected patients who might have concurrent bacterial pneumonia [13]. Our study showed that leukocytosis and neutrophilia were notably higher in bacterial coinfected patients. This finding could alert physicians about the possibility of bacterial coinfection, as clinical diagnosis may be insufficient and bacterial cultures take time. Eighty-two percent of our patients received empiric or definitive antibiotics at some point during admission which was comparable to high rates in a China study [9].

The limitation of our study includes its' retrospective design and a small sample size which was unavoidable, as we were limited by the actual number of cases during the study period and because it was a single centre study. As such, our study was inadequately powered to examine the influence of certain characteristics. Nasopharyngeal aspirates may have questionable pathogenic role, however the 3 patients with positive NPA were treated with appropriate antibiotics as they were felt to be clinically relevant. *Mycoplasma *serology was not performed in all patients and the request was based upon physicians' discretion, this may have underestimated the actual number of cases. The preadmission antibiotic therapy could underestimate the bacterial coinfection rates. Despite these limitations, we identified bacteria coinfection in 28% of our patients.

In conclusion, our study suggests that bacterial coinfection is not uncommon in H1N1 infected patients and laboratory investigations should go beyond establishing a viral cause alone. Bacterial coinfection was more frequently seen in the older age group and was associated with higher rates of complications. As adjunct to clinical findings, clinicians need to have a high index of suspicion if neutrophilia was identified on admission as it may denote bacterial coinfection. A larger scale study will be useful to further confirm our findings.

## Competing interests

The authors declare that they have no competing interests.

## Authors' contributions

AD participated in study design, analyzed the data and wrote the manuscript. NCF participated in study design and collected the clinical data. SSH participated in study design and carried out RT-PCR. PK carried out PCR for H1N1, serology testing and culture for bacterial samples. GR participated in study design, coordinated sample collection from the wards and supervised all the laboratory diagnosis. All authors read and approved the final manuscript.
